# HIV-1 Drug Resistance Profiles of Low-Level Viremia Patients and Factors Associated With the Treatment Effect of ART-Treated Patients: A Cross-Sectional Study in Jiangsu, China

**DOI:** 10.3389/fpubh.2022.944990

**Published:** 2022-07-14

**Authors:** Defu Yuan, Ying Zhou, Lingen Shi, Yangyang Liu, Jing Lu, Jianshuang Chen, Gengfeng Fu, Bei Wang

**Affiliations:** ^1^Key Laboratory of Environmental Medicine Engineering of Ministry of Education, Department of Epidemiology and Health Statistics, School of Public Health, Southeast University, Nanjing, China; ^2^Jiangsu Provincial Center for Disease Control and Prevention, Nanjing, China

**Keywords:** low-level viremia, drug resistance, treatment effects, HIV, AIDS

## Abstract

**Objectives:**

Evaluating the drug resistance (DR) profiles of LLV patients and the influencing factors of treatment effects in Jiangsu Province.

**Method:**

The Pol gene (Reverse transcriptase and protease) was amplified and sequenced to identify the genotypes and DR profiles among LLV patients in 2021. Questionnaire survey was conducted among HIV/AIDS patients to investigate the potential influence factors of treatment effects.

**Results:**

242 Pol genes were amplified from 345 specimens, and ten genotypes were detected. The DR rate was 40.5%, with 66, 86, and 14 being resistant to NRTIs, NNRTIs, and PIs, respectively. Patients treated with the 2NRTIs+PIs regimen were detected with more DR; and drug resistance was less detected in married or cohabiting patients than unmarried patients. Non-smokers were less likely to develop LLV at follow-up than smokers; patients with stage II clinical stage at diagnosis and using 2NRTIs+PIs regimen were also more likely to develop LLV at follow-up.

**Conclusion:**

Drug resistance profiles in LLV patients are severe and differ in treatment regimens and marital statuses. Meanwhile, smoking history, clinical stage, and treatment regimen may influence the therapeutic effect. It is necessary to include LLV people in the free drug resistance testing program.

## Introduction

In 2020, about 1.5 million HIV cases were reported worldwide (1), and 62 167 AIDS cases were reported in China (2), There were 3 755 newly reported HIV/AIDS patients in Jiangsu Province, an increase of nearly 9.7% compared with last year. By the end of October, there were 35 284 living HIV/AIDS patients in Jiangsu province, among which the proportion of ART recipients had reached 99.1% (3).

ART can limit viral replication to <50 copies/ml and effectively prolong the life of HIV/AIDS patients. However, in ~10–30% of patients, standard ART does not fully control viral replication, and these patients show low levels of viral replication in their plasma, which is known as low-level viremia (LLV). According to WHO guidelines, LLV is defined as viral load (VL) between 50 and 1,000 copies/ml (4). However, LLV patients are not covered by free drug resistance monitoring in Jiangsu Province.

In previous studies that used 1,000 copies/ml as the Virological Failure (VF) criterion, Acquired Drug Resistance (ADR) was reported from 10.96 to 91.67% (5, 6), and as of 2017, the total ADR in China was 44.7% (7). Many studies pointed out that the mutation rate of drug resistance (DR) is 30–46% in LLV patients, and the highest can even reach 86% (8). However, there are few studies on drug resistance in LLV patients in China, and the drug resistance profiles of this population are still unclear.

The influencing factors of LLV and its impact on clinical prognosis remain controversial (9–15). In addition, there are few relevant research results in China, Only one long-term cohort study was conducted in Shenyang in 2020, and the results showed that baseline VL, CD4 cell counts, ethnicity, treatment duration, treatment regime, and virus subtype were all related to the occurrence of LLV, while different LLV groups and maintenance time were related to the occurrence of treatment failure (16). Nevertheless, there is still a lack of relevant research in Jiangsu Province.

In order to understand the drug resistance profiles of LLV patients in Jiangsu Province and explore possible factors related to the occurrence of LLV, this study tested the drug resistance of LLV patients who were followed up in Jiangsu province in 2021, and a questionnaire survey was also conducted among LLV patients and matched Viral Suppression (VS) patients, hoping to provide reference information for subsequent treatment effect evaluation, treatment regimen adjustment, life quality improvement, and DR strains' transmission reduction.

## Materials and Methods

### Study Design and Study Object

#### Molecular Epidemiological Investigation

A cross-sectional study design was used to study the DR profiles in LLV patients who were followed up in Jiangsu Province in 2021. Inclusion criteria included: (1) Management (treatment and follow-up) in Jiangsu Province; (2) The subjects were older than 18 years at follow-up; (3) ART time at follow-up was more than 6 months; (4) VL was in the range of 50–1,000 copies/ml during follow-up; (5) Informed consent was given to collect and use biological samples during follow-up.

#### Questionnaire Investigation

A matched case-control study design was adopted, LLV patients were selected as the case group, and matched sampling was conducted among VS patients in a 1:1 ratio according to the characteristics of included LLV patients (region, gender, and age) to carry out epidemiological case investigation. Inclusion criteria were: (1) age 18 and above; (2) HIV positive patients confirmed in Jiangsu Province; (3) The follow-up period was from January 2021 to December 2021; (4) the treatment duration was more than half a year (≥180 days) at the time of follow-up; (5) Patients in the case group were 50–1,000 copies/ml (including 50 and 1,000), while patients in the control group were VS patients (VL<50 copies/ml); (6) Voluntarily participate in the program and give informed consent.

### Sample Size Calculation of Questionnaire Survey

The results of the follow-up treatment database showed that about 20% of the patients used the treatment regimen containing Protease Inhibitors (PIs), with the treatment regimen as the primary exposure factor to be observed, setting OR = 2.7, Phi = 0.2, α = 0.05, β = 0.10. The sample size calculated by PASS (Version: 2021) software is *N* = 272. Assuming that the non-response rate is 10% and the effective rate of the questionnaire is 90%, the sample size is *N* = 272/(1–0.1)/0.9 = 336 cases (168 LLV and 168 controls, respectively), and the volume of questionnaires should be appropriately increased according to the actual situation.

### Sample Collection and Experiment Operation

The samples of this study were collected by the drug resistance monitoring program for HIV/AIDS patients in Jiangsu Province. The collected specimens were extracted by the Lizhu automatic nucleic acid extractor (4-channel). Target genes in the Pol region (Reverse transcriptase and protease) were amplified by the in-house method. Reaction conditions, amplification system, and primers are shown in Table 1. Gel electrophoresis was performed on the amplified products, and a gel imager (Tanon-3500B) was used to determine the electrophoresis results. PCR products were sent to Sangon Biotech for sequencing.

**Table 1:** Amplification system of Pol gene.

	**Amplification stage**	
	**RT-PCR (25 μL)**	**Nest-PCR (50 μL)**
Reaction conditions	50°C, 30 min	94°C, 2 min
	94°C, 3 min	54°C, 1 min
	35 cycles (94°C, 30 s; 54°C, 30 s; 72°C, 1 min 30 s)	72°C, 1 min 30 s
	72°C, 10 min	30 cycles ( 94°C, 30 s; 54°C, 45 s; 72°C, 45 s)
	4°C, Constant preservation	72°C, 10 min
		4°C, Constant preservation
Reagent (volume: μL)	2 × One step mix (12.5)	2 × Taq Plus Master Mix II (Dye Plus) (25)
	RT-21 (1)	RT-20 (2)
	MAW26 (1)	Pro-1 (2)
	RNase-free ddH2O (4.25)	RNase-free ddH2O (16)
	One Step Enzyme Mix (1.25)	Template DNA (5)
	Template RNA (5)	
Primers	MAW26 (TTGGAAATGTGGAAAGGAAGGAC, HXB2: 2028-2050)	Pro-1 (CAGAGCCAACAGCCCCACCA, HXB2: 2147-2166)
	RT21 (CTGTATTTCTGCTATTAAGTCTTTTGATGGG, HXB2: 3509-3539)	RT20 (CTGCCAGTTCTATGCTTC, HXB2: 3441-3462)

### Baseline Data Collection and Questionnaire Survey

The Ethics Review Committee of Jiangsu Provincial Center for Disease Control and Prevention has approved this study (Approval No: JSJK2021-B017-01). All the included patients' baseline data, follow-up data, and laboratory test data during treatment were obtained from the baseline information database and treatment information database of HIV patients in Jiangsu Province, and a supplementary information survey was conducted by online questionnaire and offline interview.

### Data Management and Analysis

#### Sequence Processing and Subtype Determination

ChromasPro (Version: 1.6) was used for sequence spliced, website (https://comet.Lih.lu/) and HIV database (https://www.hiv.lanl.gov/content/sequence/BASIC_BLAST/basic_blast.html) were used for subtype determination. FastTree (Version: 1.4.3) was used to construct the phylogenetic tree to verify the online subtype determination results.

#### Drug Resistance Analysis

Pol region sequences were uploaded to the HIV Drug Resistance Database (https://hivdb.Stanford.edu/) for drug resistance analysis. The drug resistance degree of each drug was divided into five grades: sensitive (1 score), potential resistance (2 score), low-level resistance (3 score), moderate-level resistance (4 score), and high-level resistance (5 score). In this study, samples with sensitive and potential resistance were regarded as not resistant, while those with low or above level resistance grade (resistance score ≥3) were regarded as resistant. Drug resistance rate = number of drug resistance ÷ (number of drug resistance + number of non-drug resistance) * 100.

### Statistical Analysis

Excel was used to manage the collected information, and SPSS (Version: 25) was used for statistical analysis. Quantitative data were described by mean ± standard deviation (X ± S), and comparison between groups was performed by two-sample independent *T*-test or analysis of variance. Qualitative data were described by ratio or constituent ratio. χ2 test, Fisher's exact probability method, or Kruskal-Wallis method were used for statistical analysis. Logistic regression model was used for univariate and multivariate analysis, and the α = 0.05.

## Results of Molecular Epidemiological Investigation

### Characteristics of LLV Patients

In this study, a total of 345 samples were collected throughout 2021. After nucleic acid extraction, PCR amplification and sequencing were performed on the plasma of 345 samples, Pol genes of 255 patients were obtained. Thirteen cases of sequences with poor quality or unmatched information were removed, and 242 cases of sequences meeting the research requirements were finally included. The success rate of amplification was 70.14% (VL 50–199: 59.3%; VL 200–499: 79.4%; VL: 500–1,000: 91.1%). The characteristics of the successfully amplified subjects are shown in Table 2.

**Table 2:** Characteristics of LLV patients.

**Characters**	***N***	**Number of patients (%)**		**χ^2^**	***P*-value**
		**Drug sensitive**	**Drug resistance**		
**Gender**				0.82	0.36
Men	213	129 (60.6)	84 (39.4)		
Women	29	15 (51.7)	14 (48.3)		
**Age**				4.27	0.37
<25	11	5 (45.5)	6 (54.5)		
25–34	65	33 (50.8)	32 (49.2)		
35–44	41	26 (63.4)	15 (36.6)		
45–54	50	32 (64.0)	18 (36.0)		
≥55	75	48 (64.0)	27 (36.0)		
**Marital status**				6.97	0.03*
Unmarried	73	38 (52.1)	35 (47.9)		
Married or cohabiting	133	89 (66.9)	44 (33.1)		
Divorced or widowed	36	17 (47.2)	19 (52.8)		
**Sample source**				2.83	0.24
Southern Jiangsu	118	68 (57.6)	50 (42.4)		
Central Jiangsu	55	38 (69.1)	17 (30.9)		
Northern Jiangsu	69	38 (55.1)	31 (44.9)		
**Virus load (copies/ml)**				6.75	0.03*
50–199	137	91 (66.4)	46 (33.6)		
200–499	54	29 (53.7)	25 (46.3)		
500–1,000	51	24 (47.1)	27 (52.9)		
**Treatment duration (year)**				2.90	0.41
0~	33	22 (66.7)	11 (33.3)		
1~	38	24 (63.2)	14 (36.8)		
2~	51	33 (64.7)	18 (35.3)		
3~	120	65 (54.2)	55 (45.8)		
**CD4 cell counts (cells/μL)**				2.86	0.24
<200	47	23 (48.9)	24 (51.1)		
200–499	115	70 (60.9)	45 (39.1)		
≥500	80	51 (63.8)	29 (36.3)		
**Treatment regimens**				11.96	0.01*
2NRTIs+NNRITs	159	103 (64.8)	56 (35.2)		
2NRTIs+INSTIs	14	9 (64.3)	5 (35.7)		
2NRTIs+PIs	44	16 (36.4)	28 (63.6)		
Others	25	16 (64.0)	9 (36.0)		
**Clinical stages**				4.89	0.18
I	107	60 (56.1)	47 (43.9)		
II	43	32 (74.4)	11 (25.6)		
III	47	26 (55.3)	21 (44.7)		
IV	45	26 (57.8)	19 (42.2)		
Total	242	144	98		

*NRTIs, Nucleoside Reverse Transcriptase Inhibitors; NNRTIs, Non-nucleoside Reverse Transcriptase Inhibitors; INSTIs, Integrase Strand Transfer Inhibitors; PIs, Protease Inhibitors*.

*P < 0.05.

Chi-square test results showed that there were differences in drug resistance detection rates among patients with different marital statuses, viral load, and treatment regimens (Table 2). The detection rate of drug resistance was lower (33.1%) in married or cohabiting patients and lower in patients with VL 50–199 copies/ml (33.6%), while more patients with drug resistance were found in the VL 500–1,000 copies/ml group (52.9%). Resistance detection rates were also lower in patients treated with 2NRTIs+NNRTIs and 2NRTIs+INSTIs (35.2 and 35.7%, respectively) than in patients treated with 2NRTIs+PIs (63.6%).

### Distribution of HIV-1 Genotypes in LLV Patients

**Figure 1:**
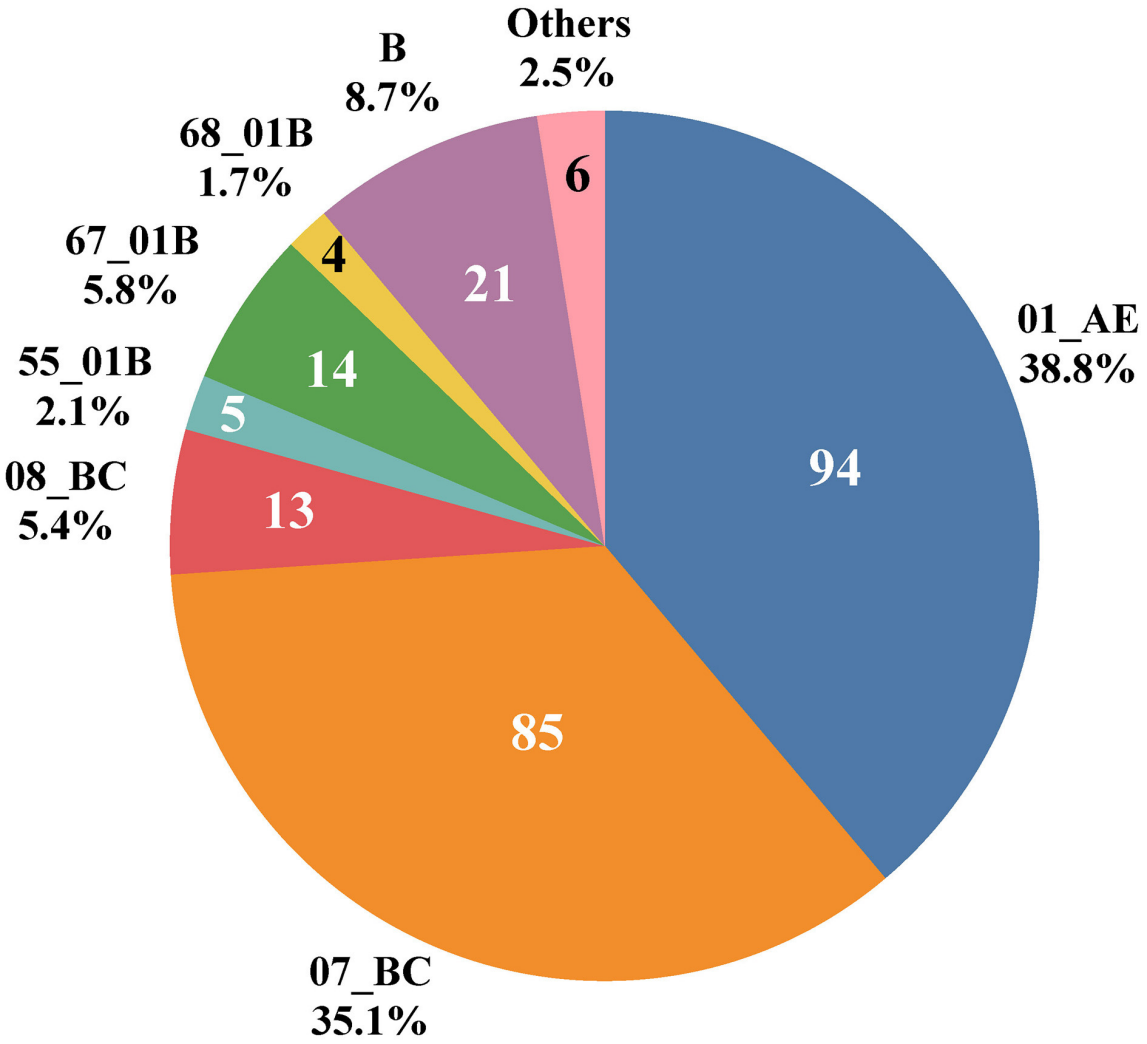
HIV-1 genotypes distribution among 242 LLV patients in Jiangsu Province, China.

**Figure 2:**
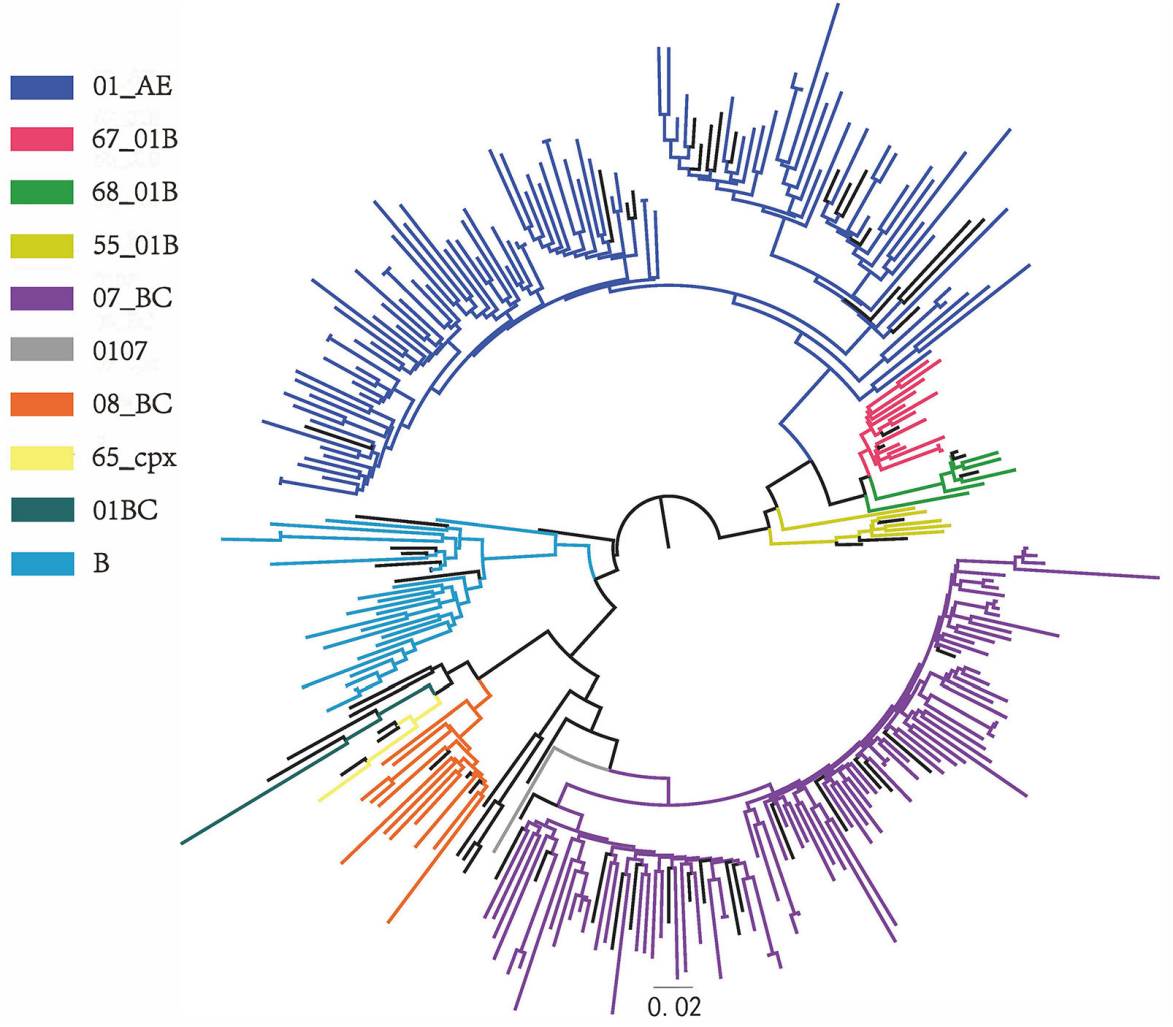
Phylogenetic tree. Phylogenetic tree was constructed from 242 pol gene sequences of LLV patients in Jiangsu province and reference sequences by FastTree software, with parameters as GTR + G + I replacement model and Bootstrap value ≥0.90.

Ten HIV-1 genotypes were detected based on the Pol gene fragments from 242 cases (Figure 1). A total of 10 HIV-1 genotypes were detected, the proportion from high to low were as follows: Circulating Recombinant Forms (CRF) 01_AE (94, 38.8%), CRF07_BC (85, 35.1%), B (21, 8.7%), CRF67_01B (14, 5.8%), CRF08_BC (13, 5.4%), CRF55_01B (5, 2.1%), CRF68_01B (4, 1.7%), 01BC (4, 1.7%), 0107 (1, 0.4%) and CRF65_cpx (1, 0.4%). The genotypes detected were mainly CRF with a small amount of Unique Recombinant Forms (URFs) (01BC and 0107). Figure 2 shows the phylogenetic tree constructed with Pol genes.

### Drug Resistance Characteristics in LLV Patients

#### Frequency Analysis of Different Types of Drug Resistance

**Figure 3:**
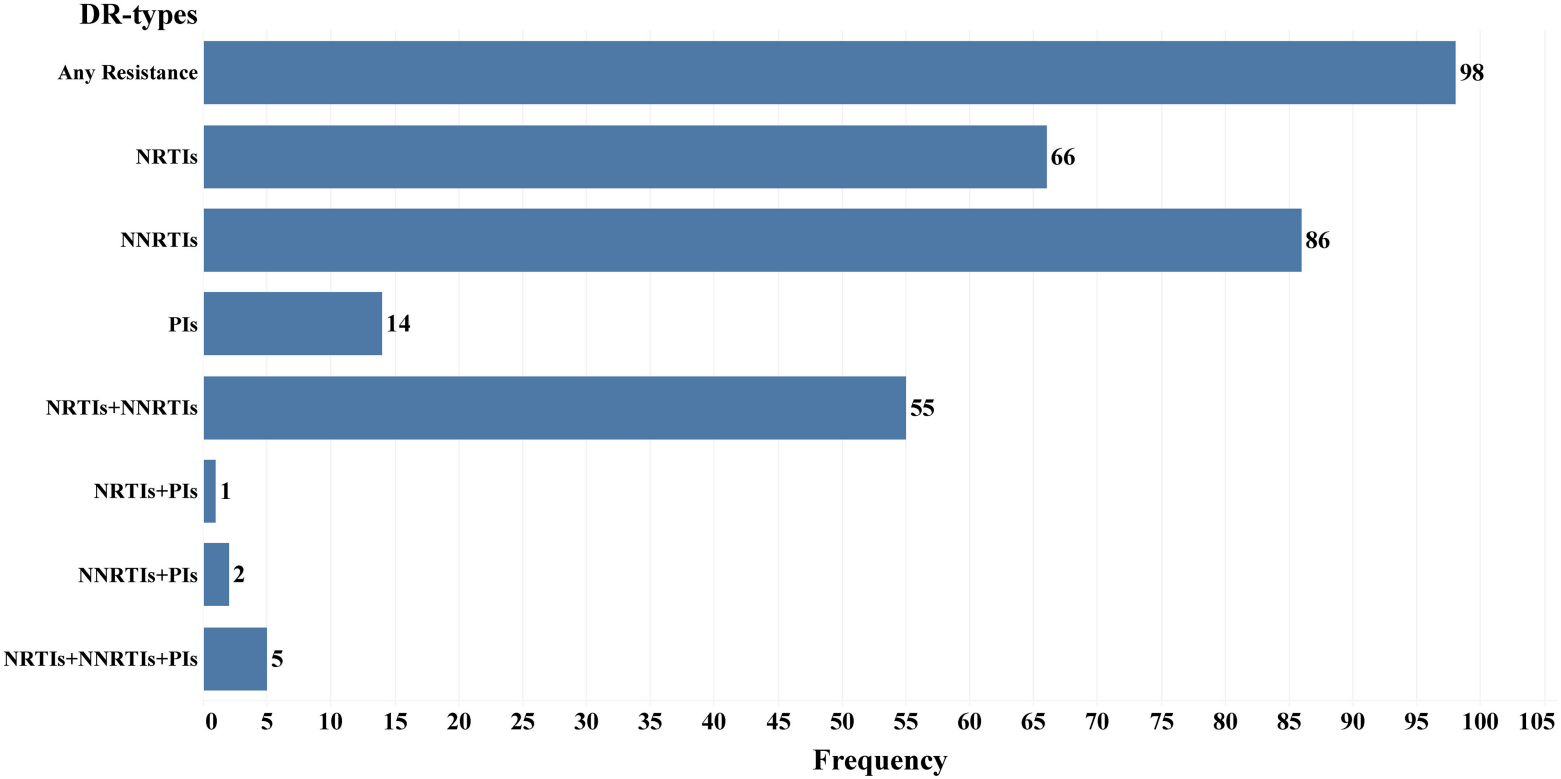
Frequency of different drug resistance types in 98 Drug resistance LLV patients in Jiangsu Province, China.

There are 98 drug resistance sequences among 242 pol genes (low or above level resistance to any class of drugs), and the drug resistance rate was 40.5% (98/242). Among them, 66 (67.3%), 86 (87.8%), and 14 (14.3%) were resistant to NRTIs, NNRTIs, and PIs, respectively. A total of 63 samples showed low or above level drug resistance to more than one class of drugs, including 55 cases of simultaneous resistance to NRTIs and NNRTIs, 1 case of simultaneous resistance to NRTIs and PIs, 2 cases of simultaneous resistance to NNRTIs and PIs, and 5 cases of drug resistance to all three types of drugs (Figure 3).

#### Influencing Factors of Drug Resistance in LLV Patients

Using drug resistance profiles as the dependent variable and multiple factors as independent variables, univariate regression analysis showed statistically significant differences among marital status (Married or cohabiting vs. Unmarried: OR = 0.537; 95%CI: 0.299–0.963; *P* = 0.037) and treatment regimens (2NRTIs+PIs vs. 2NRTIs+NNRITs: OR = 3.219; 95%CI: 1.606–6.450; *P* = 0.001), multivariate regression analysis was further performed with marital status and treatment regimen as independent variables (Table 3), and the results showed that compared with unmarried patients, drug resistance was less detected in married or cohabiting patients (OR = 0.509, 95%CI: 0.279–0.928). Compared with the 2NRTIs+NNRTIs regimen, more drug resistance was detected in patients treated with the 2NRTIs+PIs regimen (OR = 3.240; 95%CI: 1.595–6.584).

**Table 3:** Factors associated with drug resistance among LLV patients.

**Characters**	**Univariate Regression**		**Multivariate Regression**	
	**OR (95%CI)**	***P***	**OR (95%CI)**	***P***
**Marital status**				
Unmarried	Ref		Ref	
Married or cohabiting	0.537 (0.299–0.963)	0.037*	0.509 (0.279–0.928)	0.028*
Divorced or widowed	1.213 (0.546–2.699)	0.635	1.034 (0.450–2.374)	0.938
**Treatment regimens**				
2NRTIs+NNRITs	Ref		Ref	
2NRTIs+INSTIs	1.022 (0.327–3.197)	0.970	1.108 (0.347–3.540)	0.862
2NRTIs+PIs	3.219 (1.606–6.450)	0.001*	3.240 (1.595–6.584)	0.001*
Others	1.035 (0.430–2.492)	0.940	1.149 (0.470–2.810)	0.761

^*^P < 0.05.

#### Characteristics of Drug Resistance Mutations

According to the drug resistance analysis results, 66 drug resistance mutation (DRM) sites were detected in 98 patients with drug resistance. Fifty-four DRM sites were detected in the reverse transcription (RT) region, including 25 NRTIs related sites with 125 mutations. M184V mutation frequency was the most frequent (40.8%, 51/125), followed by K65R (12.0%, 15/125). There were 29 DRM sites associated with NNRTIs, with 184 mutations occurring in total, mainly K103N (24.5%, 45/184) and Y181C (8.2%, 15/184), followed by K101E (6.0%, 11/184) and V106M (6.0%, 11/184).

**Figure 4:**
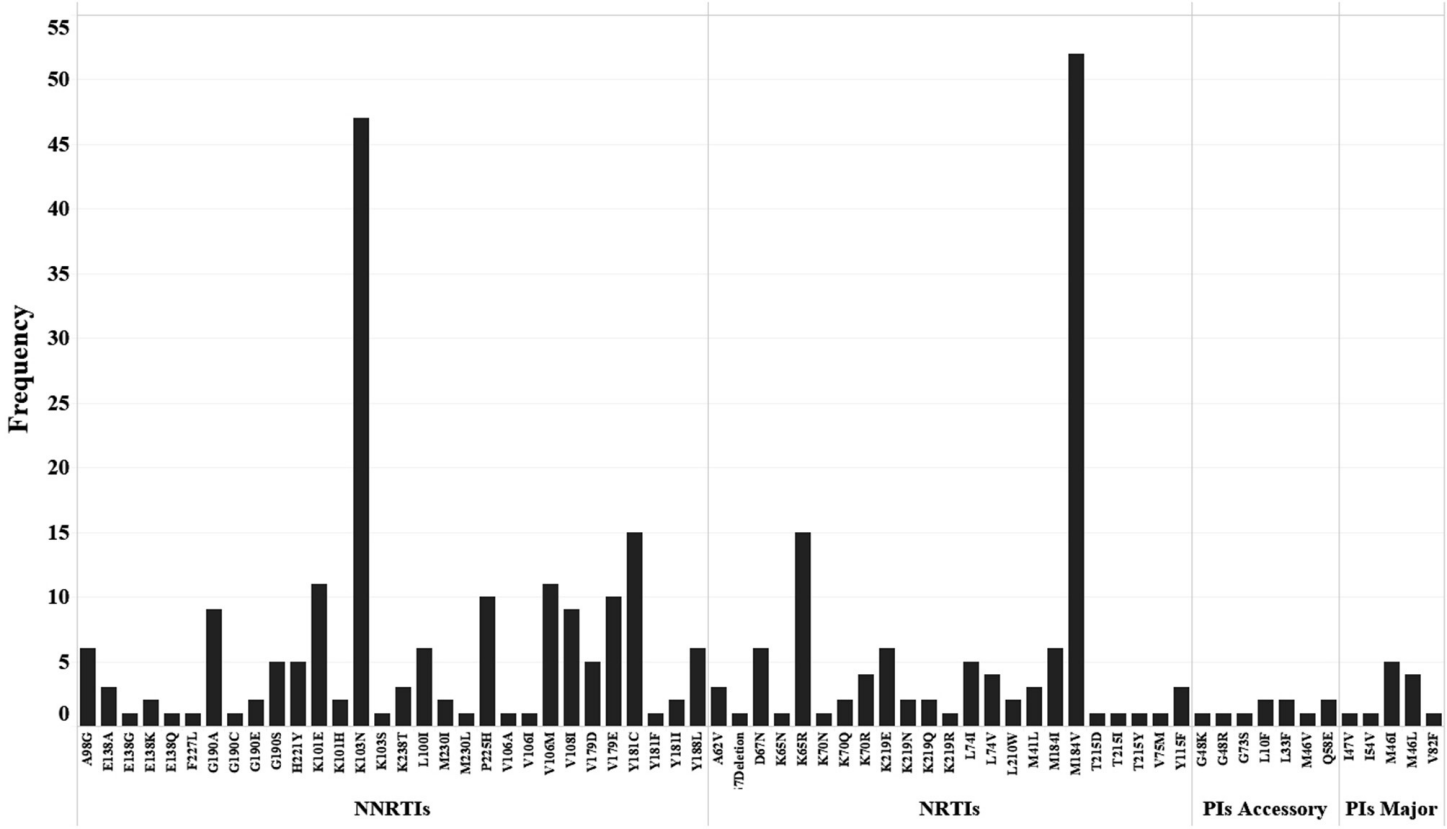
Distribution and frequency of drug resistance mutation sites to NNRTIs, NRTIs and PIs (Major and Accessory).

A total of 12 DRM sites were detected in the protease inhibitors (PIs) region, among which 5 were the major mutation sites, resulting in 11 mutations, with M46I (45.5%, 5/11) and M46L (27.3%, 3/11) having the highest frequency. The frequency and percentage of other DRM are shown in Figure 4.

#### The Influence of DRM Sites on Drug Sensitivity

The DRM sites and corresponding drug sensitivity of 98 patients with drug resistance are shown in the Supplementary Materials. Sixty-six of the 98 patients had NRTIs-related DRM sites, and all caused low or above level drug resistance, 37 of them carried a single DRM site, accounting for 56.1%, and the rest all carried two or more NRTIs DRM sites, which mainly c aused medium or high-level resistance to Abacavir (ABC), Emtricitabine (FTC), and Lamivudine (3TC).

Ninety patients developed NNRTIs-related DRM sites, 86 patients developed low or above level drug resistance to NNRTIs drugs, and 56 patients carried two or more DRM sites (62.2%). Except for four patients with only V179D/E mutation sites who did not develop DR to NNRTIs drugs, the rest had medium or high-level resistance to Efavirenz (EFV) and Nevirapine (NVP). Meanwhile, more than half of the patients had medium or high-level resistance to Doravirine (DOR) and Rilpivirine (RPV) (64.0%, 55/86).

Seventeen patients developed DRM sites associated with PIs drugs, and 14 patients had low or above level resistance, of which 71.4% carried only a single mutation site (10/14), mainly producing low or medium-level resistance to Nelfinavir (NFV).

#### Drug Resistance Profiles in Patients With Different Treatment Regimens

The treatment regimens were divided into four categories (Table 4). Patients using the 2NRTIs+NNRTIs regimen accounted for the highest proportion (57.1%, 56/98), with 39 patients resistant to NRTIs, ABC, FTC, and 3TC having the highest drug resistance frequency; 49 patients resistant to NNRTIs, and all were resistant to EFV and NVP. Patients using the 2NRTIs+PIs regimen accounted for 28.6% (28/98); among patients using this regimen, there were 20 and 25 patients resistant to NRTIs and NNRTIs, respectively, but only three patients were resistant to PIs.

**Table 4:** Drug resistance profiles in patients with different treatment regimens.

**Types**	**Drug**	**Treatment Regimens**				**Total**
		**2NRTIs+NNRTIs**	**2NRTIs+PIs**	**2NRTIs+INSTIs**	**Others**	
NRTIs	ABC	38	20	1	6	65
	AZT	4	1	0	2	7
	D4T	16	7	0	2	25
	DDI	20	10	0	3	33
	FTC	38	20	1	5	64
	3TC	38	20	1	5	64
	TDF	14	7	0	2	23
	DR cases	39	20	1	6	
NNRTIs	DOR	31	17	2	5	55
	EFV	49	25	4	8	86
	ETR	21	13	0	2	36
	NVP	49	25	4	8	86
	RPV	28	16	2	4	50
	DR cases	49	25	4	8	
PIs	ATV	0	0	2	0	2
	DRV	0	0	1	0	1
	FPV	1	0	3	0	4
	IDV	2	0	3	0	5
	LPV	1	0	3	0	4
	NFV	7	1	3	1	12
	SQV	1	0	2	0	3
	TPV	3	0	1	0	4
	DR cases	9	3	1	1	

*ABC, Abacavir; AZT, Zidovudine; D4T, Stavudine; DDI, Didanosine: FTC, Emtricitabine; 3TC, Lamivudine; TDF, Tenofovir; DOR, Doravirine; EFV, Efavirenz; ETR, Etravirine; NVP, Nevirapine; RPV, Rilpivirine; ATV, Atazanavir; DRV, Darunavir; FPV, Fosamprenavir; IDV, Indinavir; LPV, Lopinavir; NFV, Nelfinavir; SQV, Saquinavir; TPV, Tipranavir*.

## Questionnaire Survey Results

### Characteristics of Research Object

Four hundred seventy-two questionnaires were sent out, among which nine people refused the survey. Four hundred sixty-three questionnaires were returned with a recovery rate of 98.1%. The 17 questionnaires that did not fill in survey numbers and codes as required and whose information could not match were removed, and 446 questionnaires were available. Finally, 380 questionnaires (190 LLV patients and 190 matched VS patients) were analyzed.

Most VS and LLV patients were married or cohabiting (56.3 and 52.1%), and most VS and LLV patients did not smoke or drink alcohol. Most patients were in clinical stage I at the time of diagnosis (VS: 57.9%; LLV: 41.6%), and more than half of VS and LLV patients received ART for more than 3 years (59.5 and 56.8%). The proportion of the CD4 cell count group was from high to low: 200–499 cells/μL (VS: 48.4%; LLV: 51.6%), 500 cells/μL or more (VS: 38.9%; LLV: 32.6%) and 200 cells/μL or less (VS: 12.6% and 15.8%); In VS patients, 92.6% did not miss medication in the last month, and 85.3% in LLV patients; The majority of patients received 2NRTIs+NNRTIs at follow-up (VS: 83.7%; LLV: 68.9%), followed by 2NRTIs+PIs (VS: 10.0%; LLV: 20.5%) (Table 5).

**Table 5:** Characteristics of surveyed HIV/AIDS patients.

**Characters**	***N***	**Number of patients (%)**	
		**VS**	**LLV**
**Registered residence**			
Rural	244	122 (64.2)	122 (64.2)
City	136	68 (35.8)	68 (35.8)
**Education degree**			
Illiteracy	9	5 (2.6)	4 (2.1)
Primary school	61	29 (15.3)	32 (16.8)
Junior high school	116	51 (26.8)	65 (34.2)
High school	77	46 (24.2)	31 (16.3)
Bachelor degree or above	117	59 (31.1)	58 (30.5)
**Occupation**			
Farming	76	40 (21.1)	36 (18.9)
Migrant workers	94	41 (21.6)	53 (27.9)
Employee	109	61 (32.1)	48 (25.3)
Government agencies	12	8 (4.2)	4 (2.1)
Self-employed	36	19 (10.0)	17 (8.9)
Others	53	21 (11.1)	32 (16.8)
**Marital status**			
Married or cohabiting	206	107 (56.3)	99 (52.1)
Unmarried	100	49 (25.8)	51 (26.8)
Divorced or widowed	74	34 (17.9)	40 (21.1)
**Income (RMB/month)**			
Below 1,000	71	27 (14.2)	44 (23.2)
1,000–4,999	192	102 (53.7)	90 (47.4)
5,000–9,999	99	51 (26.8)	48 (25.3)
10,000 and above	18	10 (5.3)	8 (4.2)
**Smoking History**			
Yes	102	41 (21.6)	61 (32.1)
No	278	149 (78.4)	129 (67.9)
**Drinking History**			
No	210	99 (52.1)	111 (58.4)
Once a month or less	86	51 (26.8)	35 (18.4)
2–3 times a month	56	24 (12.6)	32 (16.8)
More than once a week	28	16 (8.4)	12 (6.3)
**Treatment duration (year)**			
0~	48	27 (14.2)	21 (11.1)
1~	51	24 (12.6)	27 (14.2)
2~	60	26 (13.7)	34 (17.9)
3~	221	113 (59.5)	108 (56.8)
**CD4 cell counts (cells/μL)**			
<200	54	24 (12.6)	30 (15.8)
200–499	190	92 (48.4)	98 (51.6)
≥500	136	74 (38.9)	62 (32.6)
**Clinical stages**			
I	189	110 (57.9)	79 (41.6)
II	90	40 (21.1)	50 (26.3)
III	56	23 (12.1)	33 (17.4)
IV	45	17 (8.9)	28 (14.7)
**Spouse/partners' HIV status**			
Positive	60	26 (13.7)	34 (17.9)
Negative	200	108 (56.8)	92 (48.4)
Unclear	120	56 (29.5)	64 (33.7)
**Medication omission last month**		
Yes	42	14 (7.4)	28 (14.7)
No	338	176 (92.6)	162 (85.3)
**STD history**			
Yes	13	4 (2.1)	9 (4.7)
No	367	186 (97.9)	181 (95.3)
**Treatment regimens**			
2NRTIs+NNRITs	290	159 (83.7)	131 (68.9)
2NRTIs+INSTIs	10	3 (1.6)	7 (3.7)
2NRTIs+PIs	58	19 (10.0)	39 (20.5)
Others	22	9 (4.7)	13 (6.8)
Total	380	190	190

At the same time, considering differences in usage and dosage among treatment regimens, which might affect patients' compliance, the treatment regimens were stratified, drug omissions in a recent month were compared, and no statistical difference was found (χ^2^ = 5.133, *P* = 0.162).

### Influencing Factors of Treatment Effect in HIV/AIDS Patients

With treatment effect (LLV or VS at follow-up) as the dependent variable, univariate logistic regression was performed with other independent variables (Table 6). There were statistically significant differences among self-assessment compliance (*P* = 0.033), treatment regimen (2NRTIs+PIs vs. 2NRTIs+NNRITs: *P* = 0.003), smoking history (No smoking vs. Smoking: *P* = 0.021), clinical stage at diagnosis (Stage II vs. Stage I: *P* = 0.032; Stage III vs. Stage I: *P* = 0.025; Stage IV vs. Stage I: *P* = 0.015), and medication omission in the last month (*P* = 0.024).

**Table 6:** Factors associated with treatment effect among HIV/AIDS patients.

**Characters**	**Univariate regression**		**Multivariate regression**	
	**OR (95%CI)**	***P***	**OR (95%CI)**	***P***
**Self-assessment compliance**	0.963 (0.930–0.997)	0.033*	-	-
Treatment regimens				
2NRTIs+NNRITs	Ref		Ref	
2NRTIs+INSTIs	2.832 (0.718–11.169)	0.137	2.533 (0.609–10.533)	0.201
2NRTIs+PIs	2.491 (1.374–4.517)	0.003*	2.171 (1.152–4.093)	0.016*
Others	1.753 (0.727–4.230)	0.212	1.173 (0.454–3.028)	0.742
**Smoking history**				
Yes	Ref		Ref	
No	0.582 (0.367-0.922)	0.021*	0.579 (0.358–0.936)	0.026*
**Clinical stages**				
I	Ref		Ref	
II	1.741 (1.049–2.888)	0.032*	1.700 (1.009–2.864)	0.046*
III	1.998 (1.090–3.661)	0.025*	1.864 (0.988–3.516)	0.054
IV	2.293 (1.175–4.474)	0.015*	1.727 (0.835–3.573)	0.141
**Medication omission last month**			
Yes	Ref		-	-
No	0.460 (0.234–0.905)	0.024*	-	-

^*^P < 0.05.

Independent variables with significant univariate analysis results were included in multivariate logistic regression analysis, and no correlation was found between LLV and self-assessment compliance or medication omission (*P* > 0.05). Smoking history (No smoking vs. Smoking: *P* = 0.026), clinical stage at diagnosis (Stage II vs. Stage I: *P* = 0.046), and treatment regimen (2NRTIs+PIs vs. 2NRTIs+NNRITs: *P* = 0.016) showed statistically significant differences in treatment effect among different groups. Non-smokers were less likely to develop LLV at follow-up than smokers (OR = 0.579, 95%CI: 0.358–0.936); patients with stage II clinical stage at diagnosis (OR = 1.700, 95%CI: 1.009–2.864) and using 2NRTIs+PIs regimen (OR = 2.171, 95%CI: 1.152–4.093) were also more likely to develop LLV at follow-up (Table 6).

## Discussion

LLV patients are not included in the free drug resistance surveillance program in Jiangsu Province now. The study results showed that more than 40% of LLV patients had low or above level drug resistance to the three main ART drugs (NRTIs, NNRTIs, and PIs). Nearly 90% of the DR-patients were resistant to NNRTIs, and more than half were simultaneously resistant to NRTIs and NNRTIs. Suggesting that DR profiles in LLV patients are severe, so adjust the monitoring scope of DR and provide free DR testing for LLV patients, which can offer information for timely treatment regimen adjustment, treatment effect improving, disease progression deceleration, and patients' life quality improving, are necessary.

The information on LLV patients receiving ART shows that the elderly over 55 years old account for the highest proportion, followed by the young people between 25 and 34 years old. This may be due to the education lacking, insufficient disease understanding, poor compliance during ART, delayed diagnosis (17), other underlying diseases, and worse immune reconstitution effect is more prominent in the elderly (18). In recent years, the proportion of young people among all HIV/AIDS patients has increased, and the total number has also increased rapidly (19). In addition, most young patients have active thoughts, open sexual concepts, and unprotected homosexual behavior (20), which is at high risk of cross-infection and may also affect the treatment effect. Therefore, providing targeted consulting, testing services, and strengthening knowledge propaganda, warning education according to the characteristics of different populations can effectively improve the treatment effect and help promote prevention and control work.

Although there were no statistically significant differences in DR rates among LLV patients of different genders, DR rates were slightly higher among women. Previous studies on DR of LLV patients rarely provided detailed profiles of different genders. But previous studies on VF patients in China indicated that the DR rate of female VF patients was higher than that of males (21, 22), which was consistent with the results of this study. This may be due to higher PDR in the included female patients or because fewer female patients were collected and more resistant mutations occurred in these women. However, some studies also indicate that male VF patients have a higher drug resistance rate (23), so whether there are differences in DR rates between LLV patients of different genders needs further study.

HIV/AIDS patients in Jiangsu province mostly use the 2NRTI+NNRTIs regimen according to Chinese guidelines for diagnosis and treatment of HIV/AIDS (24). This study showed that NNRTIs resistance was the most in LLV patients, and the simultaneous resistance to NRTIs and NNRTIs was the main one among multiple-DR, same as previous results (25). More than half of DR patients developed simultaneous DR, but resistance to PIs was relatively rare. It is worth noting that this study was a cross-sectional study within 1 year; the treatment regimen of patients was identified as the one adopted at the follow-up time point, and the DR of patients may not have been generated during the use of the current regimen. However, this study did not obtain data on previous regimen changes and DR profiles of patients, so the statistical significance of the association between treatment regimens and DR in this study may not indicate the authenticity of the association. In order to verify this possibility based on existing data, we compared drug resistance profiles in patients with different regimens and found that among patients with 2NRTI+PIs regimens, only three patients developed low or medium-level DR to PIs but 25 patients developed medium or high-level resistance to NNRTIs. Although most patients are also resistant to NRTIs, it is not clear whether the resistance to NRTIs occurred before or after using the current regimen, so the difference in resistance risk between regimens still needs further study. It suggests that cross-drug resistance is severe in LLV patients; timely regimen adjustments should be made according to DR profiles, and using the PIs as the replaced drug is still a better choice, but the DR of PIs should also be monitored.

Multiple regression analysis also showed differences in the DR detection likelihood among patients with different marital statuses. DR detection likelihood in the married (Heterosexual marriage) or cohabitation (Both heterosexual and homosexual) patients was lower than that in unmarried patients; this may be due to the stable emotional state and their partner's encouragement, which promote patients' medication compliance and test timeliness, maintaining the better treatment effects, and reducing DR emergence.

Although no association between subtypes and DR was found in this study, the results showed that subtypes of drug-resistant LLV patients were mainly 01_AE and 07_BC, which had the same epidemic trend with subtypes in Jiangsu Province (26), and there were more drug-resistant mutation sites in 01_AE patients. There are few studies on the association between subtypes and DR in China; previous studies in Jiangsu province indicated that the proportion of 01_AE subtypes in the VF patients increased from 40.4% in 2009 to 56.8% in 2015 (25, 27). Meanwhile, higher X4 cell tropism (28), faster disease progression and CD4 cell counts decline (29) of 01_AE strain are also contribute to the higher DR likelihood (30, 31), which also indicates that subtype, especially 01_AE, may be related to the occurrence of drug resistance. It is suggested that DR monitoring should be strengthened in patients with these subtypes, but the differences in DR profiles among subtypes need further research.

The questionnaire survey found that patients with different smoking histories, clinical stages, and treatment regimens had different likelihoods of LLV at follow-up. Patients with smoking history were more likely to develop LLV than non-smokers at follow-up. Smoking is prevalent among HIV/AIDS patients (32) and is also considered the cause of reduced ART compliance and increased chance of complications (33, 34). Previous studies have pointed out that smoking has pro-inflammatory and immunosuppressive effects, which can change the activation of T cells (35) and affect the immune system response by changing the reactivity and differentiation of T cells (36, 37), thus damaging the immune system response to pathogens (including viruses). In addition, studies have also shown that cigarettes can induce oxidative stress and increase HIV replication (38), resulting in a higher VL in smokers than non-smokers (39). Smoking can promote HIV replication in cells has also been found *in vitro* studies (40). Although more research is needed to explore the specific cellular pathways by which cigarette smoke affects HIV replication, promoting smoking cessation and improving unhealthy lifestyles among HIV/AIDS patients may also be a supplemental measure to improving treatment effectiveness.

The WHO divides patients into four clinical stages (41). This study found that patients with different clinical stages at diagnosis have different likelihoods of LLV during subsequent treatment. Previous studies showed that patients' nutritional status recovery time in clinical stages I and II was shorter than in stages III and IV (4 vs. 8 months) (42, 43). When stages III and IV progress, the number of complications increases, the risk of infection and tumor increases (44), and the rate of weight gain is slower (45), leading to a more extended recovery period. The clinical-stage is the overall reaction of body status and influences therapeutic efficacy in various ways. Suggests that treatment regimens should combine with the baseline health condition, inhibit viral replication and cure the concomitant diseases simultaneously to improve the body's health and retard the disease process effectively.

Previous studies have pointed out that patients' compliance during treatment is related to the occurrence of LLV and is one of the main influencing factors. However, our study did not show a statistical association between compliance and treatment effect. Due to the small number of participants, the Power of negative conclusion was only 3%, so compliance cannot be considered irrelevant to the occurrence of LLV. In addition, the compliance in this study was surveyed by a self-filled questionnaire, which may be different from the actual situation, and lead to no statistical significance in the study.

Patients using PIs-based regimens were more likely to have LLV at follow-up. Cohort studies have indicated that PIs-based regimens will increase the risk of LLV in patients (46–48), and compared with PIs, patients treated with NNRTIs have a better performance in inhibiting plasma virus replication (49–51) and a lower incidence of LLV (52–55). While studies have suggested that differences between hospitals where patients recruit may influence the link between regimens and LLV, that study did not find significant differences in treatment regimens among patients from different sources (52). Leierer also noted that their results did not exclude the effect of compliance on this association (46), but Konstantopoulos still found that PIs-based regimens had nearly three times the risk of LLV compared with other regiments (56), which was consistent with the results of this study, indicating that patients using PIs-based regimens had more active virus replication and a higher possibility of virologic rebound. Viral replication is also related to different drugs' pharmacokinetics and tissue permeability. Previous studies have shown that insufficient drug concentration during ART will increase the risk of viral replication activation (57). The better pharmacokinetic characteristics of NNRTIs ensure that patients can maintain a high-level drug concentration even if poor compliance occurs (58), while the faster metabolic rate of PIs leads to a higher risk of LLV in drug omissions (59). However, further studies are needed to explore the mechanism of PIs leading to a higher risk of viral replication activation. The current drug development goals are better long-term sensitivity, longer half-life period, and fewer pharmacological interactions.

This questionnaire survey was carried out online and offline. The patients were surveyed after the management physician contacted and obtained informed consent. Hence, the cooperation degree of patients was high, the non-response rate was low, and the quality of the questionnaire was good. However, this study also has some limitations: Firstly, Among the patients included in this study, the number of patients in the VL 50–199 group was significantly higher than that in other groups, which may have biased the analysis to some extent. Secondly, due to the condition limitations, the compliance data obtained in this study were all from self-filled questionnaires (follow-up survey and supplementary survey), and lack of pharmacy re-fill records or Medication Event Monitoring System (MEMS) data, which might overestimate patients' compliance. Thirdly, the follow-up and treatment information were acquired in 2021, which was not combined with the multi-year VL variation trend and patients' PDR profiles, so the influence of factors on the treatment effect with the change of treatment duration in the whole treatment process could not be more accurately assessed. Further research and analysis are needed.

## Conclusion

This study found that nearly 40% of LLV patients in Jiangsu province had low or above level drug resistance to the ART drugs, and drug resistance profiles differed in treatment regimens and marital statuses. Meanwhile, smoking history, clinical stage, and treatment regimen may influence the therapeutic effect. Based on the existing strategy, more detailed and targeted follow-up management standards should be formulated according to different patients' characters and virus strains' molecular characteristics. In the meantime, it is necessary to include LLV people in the free drug resistance testing program and strengthen the drug resistance monitoring of ART patients to improve the treatment effect.

## Data Availability Statement

The datasets presented in this study can be found in online repositories. The names of the repository/repositories and accession number(s) can be found in the article/Supplementary Material.

## Ethics Statement

The studies involving human participants were reviewed and approved by the Ethics Review Committee of Jiangsu Provincial Center for Disease Control and Prevention (Approval No: JSJK2021-B017-01). The patients/participants provided their written informed consent to participate in this study.

## Author Contributions

GF and BW guarantee the paper's project selection, overall conduct, and correction. DY is responsible for lab operation, questionnaire collection, and article writing. YZ and LS provided help for obtaining the specimen, questionnaire collection, constructing the framework of the article, and proofreading. YL assisted with data analysis and charting. At the same time, JC and JL assisted in writing, translating, and proofreading the paper. All authors contributed to the article and approved the submitted version.

## Funding

This study was supported by Molecular Network Analysis and Social Network Exploration of HIV-1 Infection Transmission among Young Students in Jiangsu Province (0701-184160070478).

## Conflict of Interest

The authors declare that the research was conducted in the absence of any commercial or financial relationships that could be construed as a potential conflict of interest.

## Publisher's Note

All claims expressed in this article are solely those of the authors and do not necessarily represent those of their affiliated organizations, or those of the publisher, the editors and the reviewers. Any product that may be evaluated in this article, or claim that may be made by its manufacturer, is not guaranteed or endorsed by the publisher.
