# Supplementary material for: HIV-1 Drug Resistance Profiles of Low-Level Viremia Patients and Factors Associated With the Treatment Effect of ART-Treated Patients: A Cross-Sectional Study in Jiangsu, China
Source: Front Public Health. 2022 Jul 14;10:944990. doi: 10.3389/fpubh.2022.944990 (PMC9330384; doi:10.3389/fpubh.2022.944990)
Supplement: Supplementary file 1 [file Table_1.DOCX]

The Drug resistance mutation sites and corresponding drug sensitivity of 98 patients with drug resistance

| **ID** | **Subtypes** | **Drug resistance mutation sites** | | | **Drug sensitivity** | | |  |
| --- | --- | --- | --- | --- | --- | --- | --- | --- |
|  |  | **NRTI** | **NNRTI** | **PI** | **NRTI** | **NNRTI** | **PI** | |
| 1 | 01_AE | M184V | V106M,Y188L |  | ABC (L),FTC (H),LTC (H) | DOR (H),EFV (H),NVP (H),RPV (H) |  | |
| 2 | 01_AE | K65R,K70T,M184V | K101E,Y181C,  G190A,H221Y |  | ABC (H),DIT (H),DDI (H),FTC (H),LTC (H),TDF (H), | DOR (H),EFV (H),ETR (H),NVP (H),RPV (H) |  | |
| 3 | 07_BC | K65R | K101E,K103N,V106M |  | ABC (I),DIT (H),DDI (H),FTC (I),LTC (I),TDF (H) | DOR (H),EFV (H),ETR (L),NVP (H),RPV (I) |  | |
| 4 | 07_BC | D67N,K70R,  M184V,K219E | K103N,Y181C |  | ABC (H),AZT (I),DIT (I),DDI (I),  FTC (H),LTC (H),TDF (L) | DOR (L),EFV (H),ETR (I),NVP (H),RPV (I) |  | |
| 5 | 01BC |  | K103N |  |  | EFV (H),NVP (H) |  | |
| 6 | B |  | V106A |  |  | DOR (H),EFV (I),NVP (H) |  | |
| 7 | 01_AE |  | G190A |  |  | EFV (I),NVP (H),RPV (L) |  | |
| 8 | 01_AE |  |  | M46I |  |  | NFV (I) | |
| 9 | B | M184V | G190A |  | ABC (L),FTC (H),LTC (H) | EFV (I),NVP (H),RPV (L) |  | |
| 10 | 67_01B | M184V | K103N,P225H |  | ABC (L),FTC (H),LTC (H) | DOR (I),EFV (H),NVP (H) |  | |
| 11 | 55_01B | L74I,M184V | L100I,K103N,E138G,  V179E,H221Y |  | ABC (H),DDI (H),FTC (H),LTC (H) | DOR (I),EFV (H),ETR (H),NVP (H),RPV (H) |  | |
| 12 | 0107 |  |  | M46V |  |  | NFV (L) | |
| 13 | B |  | K103N |  |  | EFV (H),NVP (H) |  | |
| 14 | 07_BC | M184V | K101E,Y188L |  | ABC (L),FTC (H),LTC (H) | DOR (H),EFV (H),ETR (I),NVP (H),RPV (H) |  | |
| 15 | 68_01B |  | K103N |  |  | EFV (H),NVP (H) |  | |
| 16 | 07_BC |  | K103N |  |  | EFV (H),NVP (H) |  | |
| 17 | 07_BC | A62V,K65R,M184V | K103S,G190A |  | ABC (H),DIT (I),DDI (H),FTC (H),LTC (H),TDF (I) | EFV (H),NVP (H),RPV (L) |  | |
| 18 | B | M184V | K103N,V108I,H221Y |  | ABC (L),FTC (H),LTC (H) | DOR (I),EFV (H),NVP (H),RPV (L) |  | |
| 19 | 01_AE |  | G190A | M46L |  | EFV (I),NVP (H),RPV (L) | NFV (L) | |
| 20 | 01_AE |  | G190A | M46L |  | EFV (I),NVP (H),RPV (L) | NFV (L) | |
| 21 | 67_01B |  | K103N,V106M |  |  | DOR (I),EFV (H),NVP (H) |  | |
| 22 | 07_BC | K65R,M184V | K101E,Y181C,G190S |  | ABC (H),DIT (I),DDI (H),FTC (H),LTC (H),TDF (I) | DOR (H),EFV (H),ETR (H),NVP (H),RPV (H) |  | |
| 23 | 07_BC | M184V | V179D |  | ABC (L),FTC (H),LTC (H) |  |  | |
| 24 | 07_BC | M184V | V179D |  | ABC (L),FTC (H),LTC (H) |  |  | |
| 25 | 07_BC | M184V | V179D |  | ABC (L),FTC (H),LTC (H) |  |  | |
| 26 | 01_AE | M184V | K103N,V106M |  | ABC (L),FTC (H),LTC (H) | DOR (I),EFV (H),NVP (H) |  | |
| 27 | 08_BC | M184V | K101E,E138A |  | ABC (L),FTC (H),LTC (H) | DOR (L),EFV (L),ETR (L),NVP (I),RPV (H) |  | |
| 28 | 01_AE | M184I | Y181C,H221Y |  | ABC (L),FTC (H),LTC (H) | DOR (I),EFV (I),ETR (I),NVP (H),RPV (H) |  | |
| 29 | B | M184V | K103N,P225H |  | ABC (L),FTC (H),LTC (H) | DOR (I),EFV (H),NVP (H) |  | |
| 30 | 01_AE | K70Q,M184V | Y188L |  | ABC (I),DIT (L),DDI (L),FTC (H),LTC (H),TDF (L) | DOR (H),EFV (H),NVP (H),RPV (H) |  | |
| 31 | 08_BC | M184I | M230I | G48R  G73S | ABC (L),FTC (H),LTC (H) | DOR (L),EFV (L),ETR (L),NVP (I),RPV (I) | IDV (L)  NFV (L)  SQV (L) | |
| 32 | 01_AE | M184V | V106M,V179E |  | ABC (L),FTC (H),LTC (H) | DOR (I),EFV (H),NVP (H) |  | |
| 33 | 01_AE | A62V,K65R,M184I | K101H,Y181C,G190S |  | ABC (H),DIT (I),DDI (H),FTC (H),LTC (H),TDF (I) | DOR (H),EFV (H),ETR (H),NVP (H),RPV (H) |  | |
| 34 | 67_01B | M184V | V106M,V179E |  | ABC (L),FTC (H),LTC (H) | DOR (I),EFV (H),NVP (H) |  | |
| 35 | 01_AE | M41L,A62V,L210W,T215D | K103N,V108I |  | ABC (I),AZT (H),DIT (H),DDI (I),TDF (I) | DOR (L),EFV (H),NVP (H) |  | |
| 36 | 67_01B |  |  | M46I |  |  | NFV (I) | |
| 37 | 01_AE | M184V | K103N | I47V | ABC (L),FTC (H),LTC (H) | EFV (H),NVP (H) | FPV (I)  IDV (L)  LPV (L)  NFV (L)  TPV (I) | |
| 38 | 08_BC | K65R,M184V,K219E | L100I,Y188L |  | ABC (H),DIT (H),DDI (H),FTC (H),LTC (H),TDF (I) | DOR (H),EFV (H),ETR (I),NVP (H),RPV (H) |  | |
| 39 | 07_BC | M184V | K103N,V179E |  | ABC (L),FTC (H),LTC (H) | EFV (H),NVP (H) |  | |
| 40 | B | K65R,M184I | L100I,K103N,M230I | G48K | ABC (H),DIT (I),DDI (H),FTC (H),LTC (H),TDF (I) | DOR (I),EFV (H),ETR (I),NVP (H),RPV (H) |  | |
| 41 | 01_AE | K70N,L74I,M184V,K219R | A98G,K103N,  V108I,P225H |  | ABC (H),DIT (L),DDI (H),FTC (H),LTC (H),TDF (L) | DOR (H),EFV (H),NVP (H),RPV (L) |  | |
| 42 | B | K65R,M184I | L100I,K103N |  | ABC (H),DIT (I),DDI (H),FTC (H),LTC (H),TDF (I) | DOR (I),EFV (H),ETR (I),NVP (H),RPV (H) |  | |
| 43 | 01_AE | K65R | Y181C,G190S |  | ABC (I),DIT (H),DDI (H),FTC (I),LTC (I),TDF (H) | DOR (H),EFV (H),ETR (I),NVP (H),RPV (H) |  | |
| 44 | 01_AE | K65R | K101E,Y181C,G190S |  | ABC (I),DIT (H),DDI (H),FTC (I),LTC (I),TDF (H) | DOR (H),EFV (H),ETR (H),NVP (H),RPV (H) |  | |
| 45 | 01_AE |  | K103N,K238T |  |  | EFV (H),NVP (H) |  | |
| 46 | 01_AE | L74I,Y115F,M184V | K103N,V106I | M46I  L10F | ABC (H),DDI (H),FTC (H),LTC (H) | EFV (H),NVP (H) | FPV (L)  IDV (L)  LPV (L)  NFV (I) | |
| 47 | 07_BC | M184V | K101H,Y181C,H221Y |  | ABC (L),FTC (H),LTC (H) | DOR (I),EFV (I),ETR (I),NVP (H),RPV (H) |  | |
| 48 | 01_AE |  | Y181C |  |  | EFV (I),ETR (I),NVP (H),RPV (I) |  | |
| 49 | 07_BC |  | V106M |  |  | DOR (I),EFV (H),NVP (H) |  | |
| 50 | 07_BC | M41L,D67N,V75M,  M184V,L210W,T215Y | A98G,K101E,G190A |  | ABC (H),AZT (H),DIT (H),DDI (H),  FTC (H),LTC (H),TDF (I) | DOR (I),EFV (H),ETR (I),NVP (H),RPV (H) |  | |
| 51 | 07_BC | L74I,M184V | K103N,P225H | L33F | ABC (H),DDI (H),FTC (H),LTC (H) | DOR (I),EFV (H),NVP (H) |  | |
| 52 | 07_BC | L74I,M184V | K103N,P225H | L33F | ABC (H),DDI (H),FTC (H),LTC (H) | DOR (I),EFV (H),NVP (H) |  | |
| 53 | 55_01B | M184V | K103N,V179E |  | ABC (L),FTC (H),LTC (H) | EFV (H),NVP (H) |  | |
| 54 | 01_AE | M184V | Y181C |  | ABC (L),FTC (H),LTC (H) | EFV (I),ETR (I),NVP (H),RPV (I) |  | |
| 55 | 01_AE | K65R,M184V,K219E | K101E,G190C |  | ABC (H),DIT (H),DDI (H),FTC (H),LTC (H),TDF (I) | DOR (L),EFV (H),ETR (L),NVP (H),RPV (I) |  | |
| 56 | 01_AE | M184V | Y181I | I54V  L10F | ABC (L),FTC (H),LTC (H) | DOR (L),EFV (I),ETR (H),NVP (H),RPV (H) | ATV (L)  FPV (L)  IDV (L)  LPV (L)  NFV (I)  SQV (L)  TPV (L) | |
| 57 | 01BC | M184V |  |  | ABC (L),FTC (H),LTC (H) |  |  | |
| 58 | 01_AE | M184V | K103N,V108I |  | ABC (L),FTC (H),LTC (H) | DOR (L),EFV (H),NVP (H) |  | |
| 59 | 01_AE |  | V106M |  |  | DOR (I),EFV (H),NVP (H) |  | |
| 60 | 08_BC | D67Deletion,T69G,K70R,M184V,T215I,K219E | A98G,K103N,  V108I,P225H |  | ABC (H),AZT (H),DIT (H),DDI (H),  FTC (H),LTC (H),TDF (I) | DOR (H),EFV (H),NVP (H),RPV (L) |  | |
| 61 | 07_BC |  |  | Q58E |  |  | TPV (L) | |
| 62 | 07_BC |  | K103N,V106M |  |  | DOR (I),EFV (H),NVP (H) |  | |
| 63 | 08_BC | K70R,M184V,K219Q | L100I,K103N |  | ABC (L),AZT (I),DIT (L),DDI (L),FTC (H),LTC (H) | DOR (I),EFV (H),ETR (I),NVP (H),RPV (H) |  | |
| 64 | 07_BC | M184V | K101E,Y188L |  | ABC (L),FTC (H),LTC (H) | DOR (H),EFV (H),ETR (I),NVP (H),RPV (H) |  | |
| 65 | 07_BC | M184V | V106M,E138K,  V179D,F227L |  | ABC (L),FTC (H),LTC (H) | DOR (H),EFV (H),ETR (L),NVP (H),RPV (I) |  | |
| 66 | 01_AE | M41L,M184V | E138Q,V179E,G190A |  | ABC (L),DDI (L),FTC (H),LTC (H) | EFV (H),ETR (I),NVP (H),RPV (I) |  | |
| 67 | 07_BC | M184V | K103N | M46L | ABC (L),FTC (H),LTC (H) | EFV (H),NVP (H) | NFV (L) | |
| 68 | 01_AE | L74V,M184V | L100I,K103N |  | ABC (H),DDI (H),FTC (H),LTC (H) | DOR (I),EFV (H),ETR (I),NVP (H),RPV (H) |  | |
| 69 | 65_cpx |  | V179D |  |  | EFV (I),NVP (I),RPV (L) |  | |
| 70 | 01_AE | L74V,M184V | V108I,Y181C |  | ABC (H),DDI (H),FTC (H),LTC (H) | DOR (I),EFV (I),ETR (I),NVP (H),RPV (I) |  | |
| 71 | 01_AE | M184V | K103N |  | ABC (L),FTC (H),LTC (H) | EFV (H),NVP (H) |  | |
| 72 | 55_01B | K65N,M184V | K103N,V108I,  V179E,M230L |  | ABC (I),DIT (L),DDI (I),FTC (H),LTC (H),TDF (L) | DOR (H),EFV (H),ETR (I),NVP (H),RPV (H) |  | |
| 73 | 01_AE | K65R,K70Q | K101E,Y181C,G190S |  | ABC (H),DIT (H),DDI (H),FTC (I),LTC (I),TDF (H) | DOR (H),EFV (H),ETR (H),NVP (H),RPV (H) |  | |
| 74 | 07_BC | M184V | K103N,Y181C |  | ABC (L),FTC (H),LTC (H) | DOR (L),EFV (H),ETR (I),NVP (H),RPV (I) |  | |
| 75 | 01_AE |  | K103N |  |  | EFV (H),NVP (H) |  | |
| 76 | B | M184I |  | M46I  V82F | ABC (L),FTC (H),LTC (H) |  | ATV (I)  DRV (L)  FPV (I)  IDV (I)  LPV (I)  NFV (H)  SQV (I) | |
| 77 | 07_BC | M184V | K101E,Y188L |  | ABC (L),FTC (H),LTC (H) | DOR (H),EFV (H),ETR (I),NVP (H),RPV (H) |  | |
| 78 | 01_AE |  | K103N,K238T |  |  | EFV (H),NVP (H) |  | |
| 79 | 01_AE |  | K103N,P225H,K238T |  |  | DOR (I),EFV (H),NVP (H) |  | |
| 80 | 67_01B |  | A98G |  |  | DOR (L),EFV (L),NVP (I),RPV (L) |  | |
| 81 | 07_BC | D67N,K70R,  M184V,K219Q | K103N |  | ABC (H),AZT (I),DIT (I),DDI (I),  FTC (H),LTC (H),TDF (L) | EFV (H),NVP (H) |  | |
| 82 | B |  | K103N |  |  | EFV (H),NVP (H) |  | |
| 83 | 07_BC |  | K103N |  |  | EFV (H),NVP (H) |  | |
| 84 | 01_AE | K65R | K103N,V106M |  | ABC (I),DIT (H),DDI (H),FTC (I),LTC (I),TDF (H) | DOR (I),EFV (H),NVP (H) |  | |
| 85 | 67_01B | M184V | Y181I |  | ABC (L),FTC (H),LTC (H) | DOR (L),EFV (I),ETR (H),NVP (H),RPV (H) |  | |
| 86 | 01_AE | D67N | K103N,G190A |  | AZT (L),DIT (L) | EFV (H),NVP (H),RPV (L) |  | |
| 87 | 01_AE | K65R,D67N,L74V,  Y115F,K219N | E138A,G190E |  | ABC (H),DIT (H),DDI (H),FTC (I),LTC (I),TDF (H) | DOR (H),EFV (H),ETR (I),NVP (H),RPV (H) |  | |
| 88 | 07_BC |  | K103N,E138K |  |  | EFV (H),NVP (H),RPV (I) |  | |
| 89 | 01_AE | K65R,D67N,L74V,  Y115F,K219N | E138A,G190E |  | ABC (H),DIT (H),DDI (H),FTC (I),LTC (I),TDF (H) | DOR (H),EFV (H),ETR (I),NVP (H),RPV (H) |  | |
| 90 | 08_BC | M184V |  |  | ABC (L),FTC (H),LTC (H) |  |  | |
| 91 | B |  | K103N |  |  | EFV (H),NVP (H) |  | |
| 92 | B |  | K103N |  |  | EFV (H),NVP (H) |  | |
| 93 | 67_01B | M184V,K219E | A98G,V108I,V179E,  Y181C,P225H |  | ABC (L),DDI (L),FTC (H),LTC (H) | DOR (H),EFV (H),ETR (I),NVP (H),RPV (H) |  | |
| 94 | 67_01B | M184V,K219E | A98G,V108I,V179E,  Y181C,P225H |  | ABC (L),DDI (L),FTC (H),LTC (H) | DOR (H),EFV (H),ETR (I),NVP (H),RPV (H) |  | |
| 95 | 07_BC |  | K103N |  |  | EFV (H),NVP (H) |  | |
| 96 | 55_01B |  | V179E | M46I |  |  | NFV (I) | |
| 97 | 07_BC |  |  | Q58E |  |  | TPV (L) | |
| 98 | B |  | K103N |  |  | EFV (H),NVP (H) |  | |
